# Interactive Digital Game for Improving Visual–Perceptual Defects in Children With a Developmental Disability: Randomized Controlled Trial

**DOI:** 10.2196/34756

**Published:** 2022-04-15

**Authors:** Wen-Lan Wu, Yu-Ling Huang, Jing-Min Liang, Chia-Hsin Chen, Chih-Chung Wang, Wen-Hsien Ho

**Affiliations:** 1 Department of Sports Medicine Kaohsiung Medical University Kaohsiung Taiwan; 2 Department of Medical Research Kaohsiung Medical University Hospital Kaohsiung Taiwan; 3 Department of Physical Medicine and Rehabilitation Kaohsiung Medical University Hospital Kaohsiung Taiwan; 4 Department of Physical Medicine and Rehabilitation, School of Medicine, College of Medicine Kaohsiung Medical University Kaohsiung Taiwan; 5 Regenerative Medicine and Cell Therapy Research Center Kaohsiung Medical University Kaohsiung Taiwan; 6 Department of Healthcare Administration and Medical Informatics Kaohsiung Medical University Kaohsiung Taiwan; 7 Department of Mechanical Engineering National Pingtung University of Science and Technology Pingtung Taiwan

**Keywords:** interactive digital game, visual–perceptual defect, developmental disability, Children, Test of Visual Perceptual Skills, rehabilitation

## Abstract

**Background:**

Visual–perceptual defects in children can negatively affect their ability to perform activities of daily living. Conventional rehabilitation training for correcting visual–perceptual defects has limited training patterns and limited interactivity, which makes motivation difficult to sustain.

**Objective:**

We aimed to develop and evaluate an interactive digital game system for correcting visual–perceptual defects and evaluate its effectiveness.

**Methods:**

Participants were children aged 5 to 10 years with a diagnosis of visual–perceptual defect associated with a developmental disability. The children were randomized into a digital game group who received the traditional course of rehabilitation combined with an interactive digital game intervention (n=12) and a standard rehabilitation group (n=11) who only received the traditional course of rehabilitation. Each group underwent rehabilitation once a week for 4 weeks. Overall improvement in Test of Visual Perceptual Skills 3rd edition (TVPS-3) score and overall improvement in performance in the interactive digital game were evaluated. Parents and therapists were asked to complete a satisfaction questionnaire.

**Results:**

After 4 weeks, the TVPS-3 score had significantly increased (*P*=.002) in the digital game group (pre: mean 41.67, SD 13.88; post: 61.50, SD 21.64). In the standard rehabilitation group, the TVPS-3 score also increased, but the increase was not statistically significant (*P*=.58). Additionally, TVPS-3 score increases were significantly larger for the digital game group compared with those for the standard rehabilitation group (*P*=.005). Moreover, both parents and therapists were highly satisfied with the system. All 5 themes of satisfaction had mean scores higher than 4 in a 5-point scale questionnaire (mean 4.30, SD 0.56).

**Conclusions:**

The system has potential applications for improving visual–perceptual function in children undergoing medical rehabilitation for developmental disability.

**Trial Registration:**

ClinicalTrials.gov NCT05016492; http://clinicaltrials.gov/ct2/show/NCT05016492

## Introduction

Children with developmental disabilities often have difficulty performing activities that require well-developed visual–perceptual skills or often perform them inefficiently or with poor form. Such activities include activities of daily living that require gross motor skills or fine motor skills [1]. Children with developmental disabilities are usually referred for professional evaluation when their skill level is 20% lower than that considered to be normal for their age [2]. Parents can usually estimate the baseline ability and learning speed of children with developmental disabilities by referring to well-established childhood development standards [3,4]. Beginning therapy when the child is young—at an age when neuroplasticity is still high—can reduce future medical, educational, familial, economic, and social burdens of a developmental disability. Reducing these burdens can then enhance the dignity, adaptability, personal functional performance, and quality of life of the child and help to avoid development of emotional and psychological issues. Therefore, beginning therapy early after diagnosis of a developmental abnormality is vitally important [5].

Developmental disabilities in children are usually complicated by visual–perceptual problems. Although these problems can be improved by stimulation and training, conventional methods of visual–perceptual training have limited effectiveness because their training patterns lack extensibility, which limits the motivation, goal orientation, and sense of achievement in the learner.

The numerous scientific and technological advances in computer and digital learning technologies that have successively emerged in recent decades are currently driving broad applications of computer technology in education and therapy as well as changes in the patterns of education delivery, especially for students with special needs. Thus, researchers have begun to investigate the extent to which interactive digital games can replace traditional paper-based teaching modes.

Some research suggests that digital game designs must provide an enjoyable experience to attract users and maintain their engagement. Clinical use of multimedia for early rehabilitation and therapy for children is now very common. For example, a multimedia game used for rehabilitation may depict a skill training item as an animation of a fictitious character that provides interactive feedback, for example, dynamic acoustic-optic sounds or static images, to introduce learning concepts, provide sensory stimulation, and guide learning. The emergence of interactive digital games indeed provides clinicians with an attractive option for rehabilitation that can effectively arouse a sense of participation in the learner and sustain the interest of the learner [6-10]. Thus, to motivate learners and to stimulate their interest in the content, positive feedback and increasingly difficult skill levels should be essential elements of a multimedia digital game used as treatment to improve developmental disability. Researchers have identified therapeutic effects for multimedia digital games designed for children—improving learning in mathematics [9], find motor skill [8], or visual perception abilities [10-13] in children with autism [6], poor academic performance [7], developmental delay [8], intelligence disorder [9], attention deficit hyperactivity disorder [10], and developmental disabilities [11-13]. One of these studies [11] used the Test of Visual Perceptual Skills (TVPS) to evaluate the effectiveness of multimedia digital games for improving developmental disability but only investigated changes in a limited selection of TVPS items. Another study [12] recently reported the use of kinesthetic games for improving visual–perceptual skills in children with delayed development of these skills [13]; although the novel design of the kinesthetic games included the integration of gesture-based interaction in a digital game environment, the kinesthetic games were mainly designed to provide training in integrating visual and motor skills. Thus, Wuang et al [13] expected that the functional improvements achieved by training on the kinesthetic games would be jointly contributed by improvements in both visual attention and motor control rather than by improvements in cognitive skills alone. In order to improve cognitive skills alone, we aimed to design a digital game that provides cognitive training in the full range of TVPS items and to evaluate its feasibility for testing visual–perceptual skills.

## Methods

### Participants

**Figure 1:**
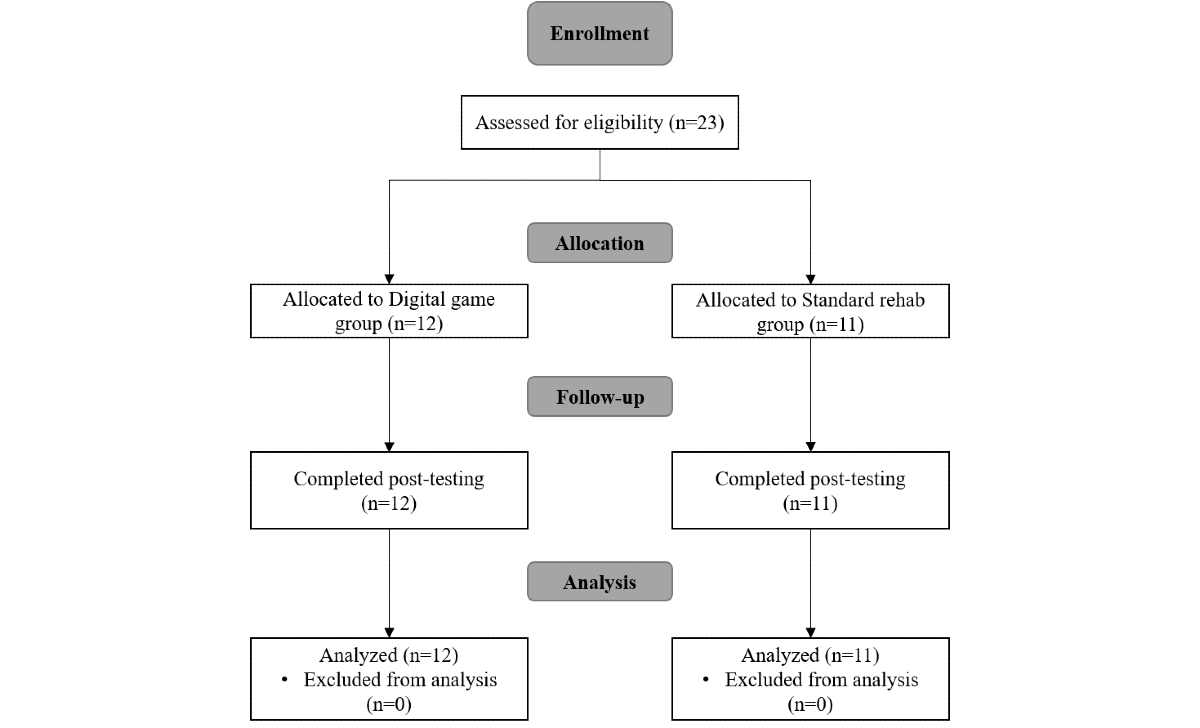
Study flowchart.

This was a prospective randomized, parallel, single-center clinical study. We recruited 23 schoolchildren with a diagnosis of visual–perceptual defect associated with a developmental disability, aged 4 to 10 years, from the pediatric rehabilitation division of a hospital in southern Taiwan (Figure 1). The enrollment criteria for this study were (1) record of developmental disability diagnosis, (2) ability to understand instructions, (3) TVPS-3 score lower than 25% of the norm reference and diagnosis of visual–perceptual defect, and (4) Test of Nonverbal Intelligence third edition score higher than 70. Participants were excluded if they (1) did not follow or did not understand the instructions for participating in the study, or if they (2) had severe defects in vision or hearing. The standard rehabilitation group (n=11) received a standard 4-week course of rehabilitation delivered in one 30-minute session per week. The digital game group (n=12) received the standard 4-week course of rehabilitation but with an additional 30-minute interactive digital game training session per week. Signed assent was obtained from all participants and signed consent was obtained from at least one parent prior to the start of the study.

### Ethics Approval

This study was approved by the institutional review board of Kaohsiung Medical University Chung-Ho Memorial Hospital (KMUHIRB-SVII-20150090).

### Visual–Perceptual Interactive Game System

The system was developed with Visual Studio (version 2015; Microsoft Inc). The system allows the training skill level difficulty to be adjusted to sustain the interest of the user.

**Figure 2:**
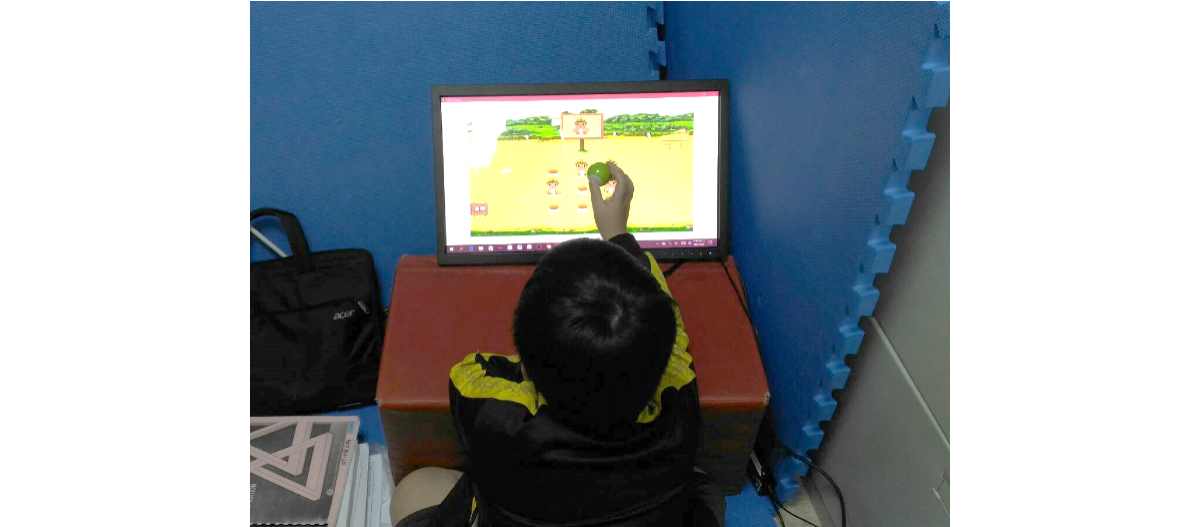
Visual–perceptual interactive digital game system.

When the game begins (Figure 2), the child is asked to hold the Bluetooth ball with the dominant hand and touch the starting position on the screen. A response signal (animation of mole) appears in the lower part of the screen, and the task is to find a mole with similar characteristics at the top of the screen. The objective of the game is to complete each task as quickly as possible, and the computer displays the average response time required to complete each task. When the game ends, the results are presented to enhance learning motivation and satisfaction in the user.

**Figure 3:**
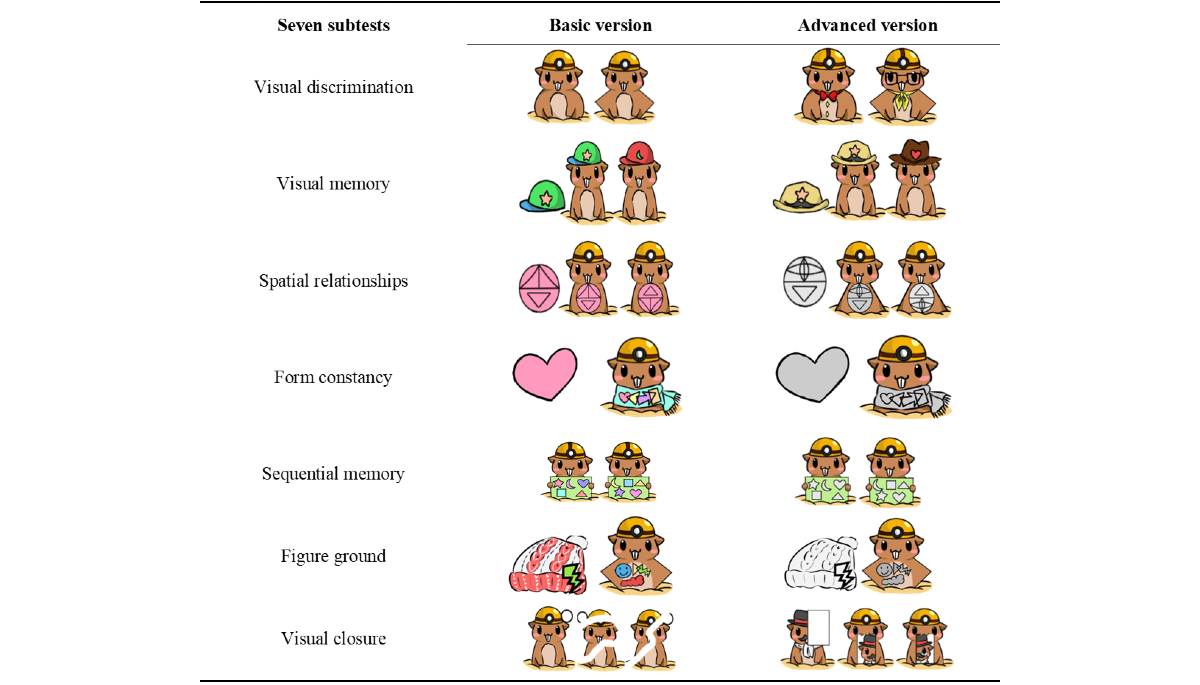
Graphics used in 7 subtests: basic version and advanced version of interactive digital game system.

After training, the responses and response times were stored in the database. Each child was required to perform 7 visual perceptual skills learning tasks, each of which was named to correspond to a TVPS-3 subscale. The game had 2 levels for each skill: basic and advanced (Figure 3). The graphics for the basic version were in color, while those for the advanced version were in black and white. The number of distractors in the advanced version was also greater than that in the basic version. Each skill level was further subdivided into primary, intermediate, and advanced. In the primary version, there was no time limit for answering each matching question or memory question. In the intermediate version, the questions were answered within a certain time limit—3 minutes for each of the questions. In the advanced version, error rate limitation was used, that is, the game ended when the number of errors exceeded 3.

The system also had a testing mode. The test difficulty was the same as the practice in the basic level of the training mode. If the user selected the testing mode, the system automatically started a sequence of 7 subtests. Each subtest had 10 questions. In accordance with the 7-item rules of the TVPS-3, 1 point was assigned to each question, for a score of 10 points for each subtest, and a total score of 70. After the test began, the user continuously answered the questions from the first test, and the test stopped if a wrong answer was given to any of the questions more than 3 times and jumped to the next test. The test scores were recorded for further analysis.

### Traditional Paper-Based TVPS-3

The TVPS is a widely used tool for clinical evaluation of visual perception [14]. The tool, which is applicable for children aged 4 to 12 years, has 7 subtests for visual discrimination, visual memory, spatial relationships, form constancy, sequential memory, figure-ground, and visual closure scoring. Each of the 7 subtests has 16 items of varying difficulty. Therefore, the test has 112 total questions. A correct response is scored as 1, and an incorrect response is scored as 0. A ceiling is reached after 3 consecutive incorrect responses on a subtest, the score recorded for the subtest is the number of questions answered correctly on the subtest so far. Raw scores are tallied for each subtest and recorded on a score sheet. Raw scores are reported as scaled scores and percentile ranks for each of the 7 subtests. The total score is reported as a percentile rank and standard score. Age-equivalent scores are provided for subtest and total scores [15].

### User Satisfaction Survey

After the experimental group had completed 4 weeks of training, a satisfaction questionnaire was administered to the parents and therapists of the children with developmental disabilities. The 20-item questionnaire content was divided into 5 themes: perceived ease of use (6 questions), perceived usefulness (4 questions), perceived joyfulness (4 questions), satisfaction (3 questions), and continued use (3 questions). We referred to the literature when designing questionnaire items for perceived ease of use [16], perceived usefulness [17], and perceived joyfulness [18]. Questionnaire items were answered on a Likert scale from 1 (completely disagree) to 5 (completely agree). A high total score indicated high satisfaction with the system. The maximum score was 100 points.

### Procedure

The TVPS-3 and interactive digital game test were carried out for the 2 groups before the intervention in the first week and the after the intervention in the fourth week of the training.

### Statistical Data Analysis

Basic characteristics and preintervention scores were compared between the 2 groups using 2-tailed independent *t* tests for interval data (age, intelligence score, and test scores) and the chi-square tests for categorical data (gender). Within-group changes in TVPS-3 and the digital game test scores were analyzed using 2-tailed paired *t* tests, and between-group differences of changes in TVPS-3 and the digital game test scores were analyzed using independent *t* tests (2-tailed). A *P* value<.05 indicated a significant difference. The Pearson correlation coefficient was calculated to verify the relevance between the digital game test score and the traditional paper TVPS-3 score. In a correlation coefficient analysis, a low correlation is defined as |*r*|<0.39; a medium correlation is defined as a |*r*|=0.40-0.69; a high correlation is defined as a |*r*|=0.70-0.99; a full correlation is defined as a |*r*|=1 [19]. The reliability of the questionnaire measurement results was assessed with Cronbach α; Cronbach α>0.70 indicated high reliability, and Cronbach α<0.35 indicated low reliability [20].

## Results

### Basic Characteristic and Preintervention Data

Children enrolled in this study had clinical diagnoses of developmental disability complicated with attention deficit hyperactivity disorder of the inattentive type (17 children), autistic spectrum disorder (1 child), speech articulation disorder (3 children), emotional disorder (1 child), and behavioral disorder (1 child). The digital game group included 10 male children and 2 female children (age: mean 7.33, SD 1.61 years; intelligence score: mean 91.50, SD 6.71 points). The standard rehabilitation group included 11 male children (age: mean 7.18, SD 1.33 years; intelligence score: mean 88.36, SD 5.78 points). There were no significant differences in gender (*P*=.16), age (*P*=.81), and intelligence score (*P*=.24) between groups. The groups did not significantly differ in preintervention scores for the TVPS-3 test (*P*=.09-.92) or for the digital game test (*P*=.15-.95).

### Comparison of Pre- and Postintervention Scores for TVPS-3 Between Groups

After training, visual discrimination (*P*=.007), visual memory (*P*=.008), form constancy (*P*=.01), sequential memory (*P*=.06), and full-test scores (*P*=.002) for the digital game group significantly increased. Scores for the remaining 3 components of the TVPS-3 were higher after the intervention, but the differences did not reach statistical significance—spatial relationships (*P*=.53), figure-ground (*P*=.08), visual closure (*P*=.06). In contrast, there were no significant changes in TVPS-3 scores for the standard rehabilitation group (*P*=.12-.73). Form constancy (*P*=.03), sequential memory (*P*=.03), visual closure (*P*=.02), and full score (*P*=.005) increases for the digital game group were greater than those for the standard rehabilitation group (Table 1).

**Table 1:** Pre- and postintervention TVPS-3 scores.

	Digital game group (n=12)			Standard rehabilitation group (n=11)			Difference (between pre- and postintervention)		
	Preintervention	Postintervention	*P* value	Preintervention	Postintervention	*P* value	Digital game group	Standard rehabilitation group	*P* value
Visual discrimination	6.50 (4.44)	9.42 (3.78)	.007	5.09 (2.17)	5.73 (2.15)	.31	2.92 (3.09)	0.64 (1.96)	.05
Visual memory	8.08 (3.99)	11.67 (3.00)	.008	5.55 (2.47)	6.27 (1.90)	.38	3.58 (3.82)	0.73 (2.61)	.05
Spatial relationships	7.33 (4.64)	8.25 (4.18)	.53	5.27 (3.38)	4.91 (3.81)	.73	0.92 (4.87)	–0.36 (3.41)	.48
Form constancy	5.75 (2.42)	8.83 (4.43)	.01	6.09 (4.39)	5.55 (4.18)	.65	3.08 (3.45)	–0.55 (3.86)	.03
Sequential memory	6.83 (3.79)	10.67 (4.36)	.006	5.09 (2.63)	5.91 (3.24)	.12	3.83 (3.90)	0.82 (1.60)	.03
Figure-ground	4.00 (1.71)	6.58 (4.38)	.08	3.91 (2.30)	4.73 (3.41)	.39	2.58 (4.68)	0.82 (2.99)	.30
Visual closure	3.17 (2.13)	6.08 (4.17)	.06	3.55 (2.54)	2.64 (1.43)	.18	2.92 (4.89)	–0.91 (2.07)	.02
Full score	41.67 (13.88)	61.50 (21.64)	.002	34.55 (13.00)	36.36 (13.40)	.58	19.83 (16.44)	1.82 (10.53)	.005

### Digital Game Test Results Before and After Intervention

For the digital game group, there were significant increases in scores for the visual discrimination component (*P*=.03) of the digital game test and the full digital game test (*P*=.01). While scores for the remaining 6 components (visual memory: *P*=.05; spatial relationships: *P*=.05; form constancy: *P*=.54; sequential memory: *P*=.76; figure-ground: *P*=.06; visual closure: *P*=.09) did not significantly differ in the digital game group, postintervention scores tended to be slightly higher than preintervention scores. In contrast, the standard rehabilitation group revealed no significant differences in digital game test scores for visual discrimination (*P*=.53), visual memory (*P*=.13), spatial relationships (*P*=.89), form constancy (*P*=.93), sequential memory (*P*=.43), figure-ground (*P*=.49), visual closure (*P*=.40), and full score (*P*=.30) (Table 2).

**Table 2:** Pre- and postintervention scores on digital game test.

	Digital game group (n=12)			Standard rehabilitation group (n=11)			Difference (between pre- and postintervention)		
	Preintervention	Postintervention	*P* value	Preintervention	Postintervention	*P* value	Digital game group	Standard rehabilitation group	*P* value
Visual discrimination	8.33 (1.72)	9.75 (0.45)	.03	8.00 (3.97)	8.73 (1.10)	.53	1.42 (2.02)	0.73 (3.74)	.58
Visual memory	7.25 (3.25)	9.08 (0.90)	.05	7.45 (3.05)	9.00 (0.77)	.13	1.83 (2.82)	1.55 (3.08)	.82
Spatial relationships	6.83 (2.79)	8.67 (2.84)	.05	5.91 (3.11)	6.09 (3.62)	.89	1.83 (2.92)	0.18 (4.14)	.28
Form constancy	8.42 (1.73)	8.67 (1.56)	.54	6.82 (3.06)	6.91 (2.07)	.93	0.25 (1.36)	0.09 (3.48)	.89
Sequential memory	6.00 (2.95)	6.25 (3.22)	.76	5.91 (3.81)	6.73 (2.49)	.43	0.25 (2.77)	0.82 (3.31)	.66
Figure-ground	5.17 (3.13)	7.50 (3.53)	.06	5.45 (3.78)	6.09 (3.02)	.49	2.33 (3.92)	0.64 (2.98)	.26
Visual closure	7.67 (2.64)	9.08 (1.38)	.09	6.45 (3.56)	7.09 (2.98)	.40	1.42 (2.68)	0.64 (2.42)	.47
Full score	49.92 (11.69)	58.92 (9.59)	.01	45.82 (19.15)	50.27 (10.13)	.30	9.00 (10.20)	4.45 (13.43)	.37

### Correlation Between TVPS-3 Test and the Digital Game Test

Spatial relationship items (*r*=0.66, *P*=.001), figure-ground items (*r*=0.65, *P*=.001), and items for the full score (*r*=0.47, *P*=.03) in the digital game test and in the TVPS-3 were significantly moderately correlated (Table 3).

**Table 3:** Analysis of Correlation between TVPS-3 Test and Digital Game Test.

	*r*	*P* value
Visual discrimination	0.06	.77
Visual memory	0.06	.78
Spatial relationships	0.66	.001
Form constancy	–0.01	.97
Sequential memory	0.25	.26
Figure-ground	0.65	.001
Visual closure	0.10	.64
Full score	0.47	.03

### Satisfaction Questionnaire

Out of 30 satisfaction questionnaires distributed to parents and therapists, 26 valid and complete questionnaires were retrieved (24 from parents and 2 from therapists). All 5 themes had mean scores higher than 4, which indicated that parents and therapists were highly satisfied with the interactive digital game system, and Cronbach α=0.89-0.96, which indicated high reliability (Table 4).

**Table 4:** Internal consistency of the satisfaction questionnaire.

	Mean (SD)	Cronbach α
Perceived ease of use	4.01 (0.71)	0.89
Perceived usefulness	4.35 (0.58)	0.91
Perceived joyfulness	4.44 (0.61)	0.95
Satisfaction	4.41 (0.58)	0.96
Continued use	4.31 (0.69)	0.96

## Discussion

Pre- and postintervention scores in the digital game group differed for all items except spatial relationships, figure-ground, and visual closure (Table 1). Previous literature has indicated that children with growth delays have low scores on visual discrimination and spatial relationships tests because insufficient visual attention and cortical visual dysfunction limits discrimination and classification capabilities [21]. Thus, these capabilities cannot be enhanced by a short period of training. In visuospatial ability training studies, verbal and visual prompts are the key factors for training success [22]. An effective digital multimedia system for learners should integrate multisensory stimulation and widely varying teaching strategies (eg, use of visual prompts such as photographs, auditory prompts, repetition) [23,24]. An example of a visual prompt is stimulus fading (ie, simultaneously presenting subjective and pictorial prompts and then fading the prompted picture in the sequence), which allows children to visually slowly concentrate on the subject or guides them to learn how to judge the subject with graphic animation paired with line changes. Moreover, so that children could more easily discriminate and judge the images, voice prompts were used to facilitate repeated training to help them learn through visualized clues [23,24].

Improvements after the digital game intervention for most TVPS-3 items were satisfactory, which is consistent with the findings of previous studies [10-13]. Multimedia provide acoustic-optic effects and interactivity, which attract the attention of learners and guides their learning. In comparison with traditional teaching materials (eg, static pictures) and traditional teaching methods (eg, paper-based activities), multimedia teaching materials can provide greater audiovisual stimuli (eg, animations and sound) to increase learning efficiency and motivation [24-27]. Additionally, a digital multimedia interface can motivate and guide the user to explore issues and accept challenges by providing highlights or keywords [28]. According to the literature [29], 60% of the learners achieve better learning outcomes when learning is supported by digital assistance multimedia. Thus, the abovementioned research results indicate that a digital multimedia program is more effective than traditional paper-based training. Integrating a digital multimedia interface in teaching can enhance learning motivation and efficiency.

There were moderate to high correlations between the TVPS-3 and digital game test (spatial relationships: *r*=0.66; figure-ground: *r*=0.65; full score: *r*=0.47) (Table 3); however, only the visual discrimination component (*P*=.03) and the full score (*P*=.01) in the digital game group exhibited significant differences between the pre- and postintervention scores. We investigated why the digital game test had lower sensitivity in detecting pre versus postintervention change compared to the TVPS-3. The digital game test was based on the 7 TVPS-3 themes (10 questions per theme and 70 total questions). The standard TVPS-3 had 16 questions per theme and 112 total questions. Therefore, the lower number of questions on the digital game test resulted in a higher rate of repetition, which decreased its sensitivity in detecting change. In addition to the high rate of repetition, the difficulty level was not high enough, since the questions in the digital game test were chosen from the primary version. Thus, digital game test questions may not have been sufficiently challenging for the identification and assessment of children with relatively higher function or older age. In future studies, the instrument should be modified so that the number of test items is identical to that of the TVPS-3, which would reduce repeatability of the image gallery and possibly enhance the credibility and validity of the system.

On the satisfaction questionnaire, 5 items had scores higher than 4 points (Table 4), which indicated that parents and therapists were highly satisfied with the interactive digital game system. The 2 clinical therapists who had participated in the course reported that the digital game could be a useful intervention for improving visual–perceptual skills in children since the operating interface was easy to use and visually attractive to children. Additionally, the improvement was comparable to that obtained from conventional paper-based game training. The 24 parents reported that their children found the digital game easy to play and found the Bluetooth controller easy to operate. As a result, their scores on the visual perception test increased, which indicates that development of visual–perceptual abilities can be enhanced when rehabilitation includes an interactive digital game assistance system [30]. Relevant literature also mentions that a digital game system that attracts the interest of users tends to have high user satisfaction and high continued use [16,17].

Additionally, traditional teaching materials are mainly paper-based. They are not easily modified and have limited modes of interactivity (mainly paper-pen mode). They cannot be shared conveniently; paper-based media can only be shared by making additional paper copies whereas digital media can be easily shared and copied via the internet. While digital multimedia has good interactivity (online synchronous learning application or operating mode combined with other virtual reality equipment), is strong in media integrity, can be flexibly adjusted at any time, can be conveniently downloaded from the cloud, and can be represented and edited with 3C software in real time [31].

In summary, the interactive digital game assistance system developed in this study has practical applications in clinical rehabilitation treatment. The system is applicable and suitable for visual–perceptual training in children with developmental disabilities.. Thus, application of this learning mode in specialized rehabilitation for children with visual–perceptual defects can substantially improve their visual–perceptual skills and can potentially increase active participation in other relevant courses of treatment.

The interactive digital game designed and developed in this study positively benefitted visual perception training for children with developmental disabilities. When used in combination with conventional training, the interactive digital game system not only confers larger improvements in vision and perception in children with developmental disabilities, it also increases their willingness to participate in the training. The interactive digital game training system developed in this study can be implemented by medical institutions, family care givers, and school educators to increase the effectiveness of conventional programs for developing visual–perceptual skills in children with developmental disabilities. To improve the applicability of the proposed system as a testing tool, our future work will expand the question bank to enable it to have a broader range of skill levels.

## Appendix

Multimedia Appendix 1 CONSORT-EHEALTH checklist (V 1.6.1).

## Abbreviations

TVPS: Test of Visual Perceptual Skills
